# 7-Chloro-4-[(7-chloro­quinolin-4-yl)sulfan­yl]quinoline dihydrate

**DOI:** 10.1107/S1600536812011087

**Published:** 2012-03-21

**Authors:** James L. Wardell, Edward R. T. Tiekink

**Affiliations:** a Centro de Desenvolvimento Tecnológico em Saúde (CDTS), Fundação Oswaldo Cruz (FIOCRUZ), Casa Amarela, Campus de Manguinhos, Av. Brasil 4365, 21040-900 Rio de Janeiro, RJ, Brazil; b Department of Chemistry, University of Malaya, 50603 Kuala Lumpur, Malaysia

## Abstract

In the title thio­ether dihydrate, C_18_H_10_Cl_2_N_2_S·2H_2_O, the *S*-bound quinolinyl residues are almost orthogonal, forming a dihedral angle of 72.36 (4)°. In the crystal, the four water mol­ecules are connected *via* an eight-membered {⋯OH}_4_ synthon with each of the four pendent water H atoms hydrogen bonded to a pyridine N atom to stabilize a three-dimensional architecture.

## Related literature

For background to the significant biological activities exhibited by quinoline derivatives, see: Natarajan *et al.* (2008 ▶). For an earlier synthesis, see: Surrey (1948 ▶).
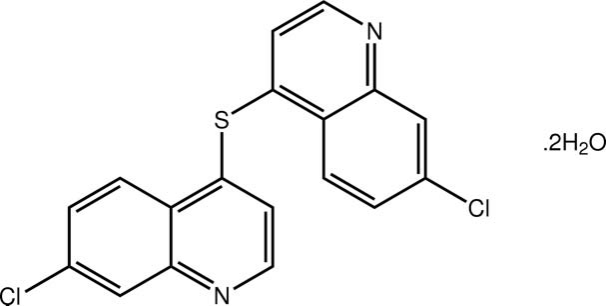


## Experimental

### 

#### Crystal data

C_18_H_10_Cl_2_N_2_S·2H_2_O
*M* *_r_* = 393.27
Monoclinic, 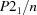

*a* = 7.8228 (2) Å
*b* = 11.5596 (3) Å
*c* = 19.2421 (13) Å
β = 97.384 (7)°
*V* = 1725.60 (13) Å^3^
*Z* = 4
Mo *K*α radiation
μ = 0.51 mm^−1^
*T* = 120 K
0.07 × 0.07 × 0.03 mm

#### Data collection

Rigaku Saturn724+ diffractometer
Absorption correction: multi-scan (*CrystalClear-SM Expert*; Rigaku, 2011 ▶) *T*_min_ = 0.930, *T*_max_ = 1.000
36518 measured reflections
3943 independent reflections
3512 reflections with *I* > 2σ(*I*)
*R*_int_ = 0.029

#### Refinement

*R*[*F* ^2^ > 2σ(*F* ^2^)] = 0.027
*wR*(*F* ^2^) = 0.076
*S* = 1.04
3943 reflections
238 parameters
6 restraints
H atoms treated by a mixture of independent and constrained refinement
Δρ_max_ = 0.46 e Å^−3^
Δρ_min_ = −0.19 e Å^−3^

### 

Data collection: *CrystalClear-SM Expert* (Rigaku, 2011 ▶); cell refinement: *CrystalClear-SM Expert*; data reduction: *CrystalClear-SM Expert*; program(s) used to solve structure: *SHELXS97* (Sheldrick, 2008 ▶); program(s) used to refine structure: *SHELXL97* (Sheldrick, 2008 ▶); molecular graphics: *ORTEP-3* (Farrugia, 1997 ▶) and *DIAMOND* (Brandenburg, 2006 ▶); software used to prepare material for publication: *publCIF* (Westrip, 2010 ▶).

## Supplementary Material

Crystal structure: contains datablock(s) global, I. DOI: 10.1107/S1600536812011087/pk2399sup1.cif

Structure factors: contains datablock(s) I. DOI: 10.1107/S1600536812011087/pk2399Isup2.hkl

Supplementary material file. DOI: 10.1107/S1600536812011087/pk2399Isup3.cml

Additional supplementary materials:  crystallographic information; 3D view; checkCIF report

## Figures and Tables

**Table 1:** Hydrogen-bond geometry (Å, °)

*D*—H⋯*A*	*D*—H	H⋯*A*	*D*⋯*A*	*D*—H⋯*A*
O1*W*—H1*W*⋯N1	0.85 (1)	2.02 (1)	2.8530 (15)	171 (2)
O1*W*—H2*W*⋯O2*W*^i^	0.84 (1)	1.94 (1)	2.7723 (14)	173 (2)
O2*W*—H3*W*⋯N2	0.85 (2)	2.01 (2)	2.8429 (14)	165 (1)
O2*W*—H4*W*⋯O1*W*^ii^	0.85 (1)	1.94 (2)	2.7683 (14)	166 (2)

Symmetry codes: (i) 
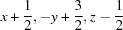
; (ii) 
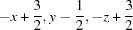
.

**Fig. 1.**
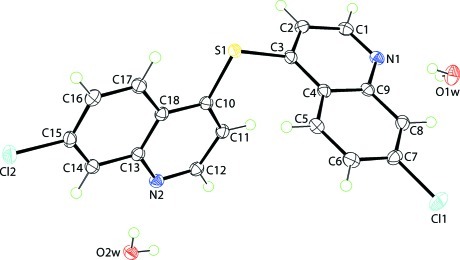
The molecular structure of (I) showing the atom-labelling scheme and displacement ellipsoids at the 50% probability level.

**Fig. 2.**
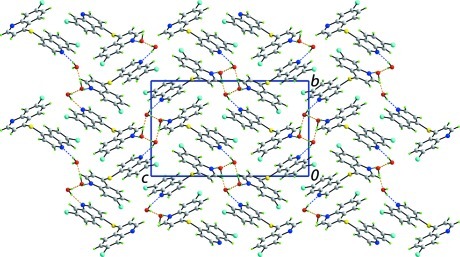
A view in projection down the a of the unit-cell contents of (I). The O—H···O and O—H···N hydrogen bonds are shown as orange and blue dashed lines, respectively.

## supplementary crystallographic information

### Comment

Interest in the title compound, bis(7-chloroquinolin-4-yl)sulfide, crystallized as a dihydrate, rests with the biological activity of related quinoline derivatives, in particular against chloroquine-resistant malaria (Natarajan *et al.*, 2008).

In (I), Fig. 1, the dihedral angle between the two quinolinyl residues [r.m.s. deviation for the 10 atoms of the N1- and N2-systems = 0.018 and 0.011 Å, respectively] of 72.36 (4)° indicates an almost orthogonal relationship.

The water molecules play a pivotal role in stabilizing the crystal structure, forming hydrogen bonds to each other and to the quinolinyl-N atoms, Table 1. The water···water interactions each to eight-membered {···OH}_4_ synthons with each pendent water-H atom hydrogen bonded to a quinolinyl-N atom to stabilize a three-dimensional architecture, Fig. 2.

### Experimental

A modification of a published procedure was adopted (Natarajan *et al.*, 2008). A solution of 4,7-dichloroquinoline (0.5 g) in EtOH (20 ml) was heated to 323 K. Thiourea (0.20 g.) was added and the mixture was stirred for 5 min. and then cooled to room temperature. The white solid was filtered off and was extracted into 0.2 *M* NaOH solution. The precipitate, bis(7-chloroquinolin-4-yl)sulfide, was collected and recrystallized from EtOH as the dihydrate; *M*.pt. 436–439 K; lit. *M*.pt: 439–440 K (Surrey, 1948).

### Refinement

The C-bound H atoms were geometrically placed (C—H = 0.95 Å) and refined as riding with *U*_iso_(H) = 1.2*U*_eq_(C). The O—H atoms were located in a difference Fourier map, and were refined with a distance restraint of O—H = 0.84±0.01 Å and with H···H = 1.39±0.03 Å; their *U*_iso_ values were constrained to 1.5*U*_eq_(O).

### Crystal data

**Table:**

C_18_H_10_Cl_2_N_2_S·2H_2_O	*F*(000) = 808
*M**_r_* = 393.27	*D*_x_ = 1.514 Mg m^−^^3^
Monoclinic, *P*2_1_/*n*	Mo *K*α radiation, λ = 0.71073 Å
Hall symbol: -P 2yn	Cell parameters from 30445 reflections
*a* = 7.8228 (2) Å	θ = 3.2–27.5°
*b* = 11.5596 (3) Å	µ = 0.51 mm^−^^1^
*c* = 19.2421 (13) Å	*T* = 120 K
β = 97.384 (7)°	Chip, colourless
*V* = 1725.60 (13) Å^3^	0.07 × 0.07 × 0.03 mm
*Z* = 4	

### Data collection

**Table:**

Rigaku Saturn724+ diffractometer	3943 independent reflections
Radiation source: Rotating Anode	3512 reflections with *I* > 2σ(*I*)
Confocal monochromator	*R*_int_ = 0.029
Detector resolution: 28.5714 pixels mm^-1^	θ_max_ = 27.5°, θ_min_ = 3.2°
profile data from ω–scans	*h* = −10→10
Absorption correction: multi-scan (*CrystalClear-SM Expert*; Rigaku, 2011)	*k* = −15→15
*T*_min_ = 0.930, *T*_max_ = 1.000	*l* = −24→24
36518 measured reflections	

### Refinement

**Table:**

Refinement on *F*^2^	Primary atom site location: structure-invariant direct methods
Least-squares matrix: full	Secondary atom site location: difference Fourier map
*R*[*F*^2^ > 2σ(*F*^2^)] = 0.027	Hydrogen site location: inferred from neighbouring sites
*wR*(*F*^2^) = 0.076	H atoms treated by a mixture of independent and constrained refinement
*S* = 1.04	*w* = 1/[σ^2^(*F*_o_^2^) + (0.0467*P*)^2^ + 0.5608*P*] where *P* = (*F*_o_^2^ + 2*F*_c_^2^)/3
3943 reflections	(Δ/σ)_max_ = 0.002
238 parameters	Δρ_max_ = 0.46 e Å^−^^3^
6 restraints	Δρ_min_ = −0.19 e Å^−^^3^

### Special details

**Table:**

Geometry. All s.u.'s (except the s.u. in the dihedral angle between two l.s. planes) are estimated using the full covariance matrix. The cell s.u.'s are taken into account individually in the estimation of s.u.'s in distances, angles and torsion angles; correlations between s.u.'s in cell parameters are only used when they are defined by crystal symmetry. An approximate (isotropic) treatment of cell s.u.'s is used for estimating s.u.'s involving l.s. planes.
Refinement. Refinement of *F*^2^ against ALL reflections. The weighted *R*-factor *wR* and goodness of fit *S* are based on *F*^2^, conventional *R*-factors *R* are based on *F*, with *F* set to zero for negative *F*^2^. The threshold expression of *F*^2^ > 2σ(*F*^2^) is used only for calculating *R*-factors(gt) *etc*. and is not relevant to the choice of reflections for refinement. *R*-factors based on *F*^2^ are statistically about twice as large as those based on *F*, and *R*- factors based on ALL data will be even larger.

### Fractional atomic coordinates and isotropic or equivalent isotropic displacement parameters (Å^2^)

**Table:**

	*x*	*y*	*z*	*U*_iso_*/*U*_eq_	
Cl1	0.85633 (5)	1.32103 (3)	0.808816 (19)	0.03066 (10)	
Cl2	−0.32119 (4)	0.87710 (3)	1.034518 (16)	0.02406 (9)	
S1	0.06139 (4)	1.02020 (3)	0.737790 (16)	0.01827 (9)	
N1	0.52489 (13)	1.07717 (9)	0.61826 (5)	0.0175 (2)	
N2	0.23604 (14)	0.80256 (9)	0.93354 (5)	0.0177 (2)	
C1	0.39198 (17)	1.01334 (11)	0.59386 (7)	0.0189 (2)	
H1	0.3906	0.9805	0.5485	0.023*	
C2	0.25041 (16)	0.99018 (11)	0.63076 (7)	0.0185 (2)	
H2	0.1584	0.9422	0.6108	0.022*	
C3	0.24849 (15)	1.03816 (11)	0.69563 (6)	0.0162 (2)	
C4	0.38920 (15)	1.10900 (10)	0.72461 (6)	0.0153 (2)	
C5	0.40017 (16)	1.16405 (11)	0.79070 (6)	0.0182 (2)	
H5	0.3077	1.1563	0.8179	0.022*	
C6	0.54164 (17)	1.22826 (11)	0.81616 (7)	0.0202 (3)	
H6	0.5484	1.2640	0.8609	0.024*	
C7	0.67699 (16)	1.24043 (11)	0.77498 (7)	0.0200 (3)	
C8	0.67128 (16)	1.19187 (11)	0.71010 (7)	0.0185 (2)	
H8	0.7634	1.2031	0.6831	0.022*	
C9	0.52619 (16)	1.12451 (10)	0.68348 (6)	0.0156 (2)	
C10	0.13671 (15)	0.93878 (10)	0.81318 (6)	0.0157 (2)	
C11	0.29263 (16)	0.88236 (11)	0.82280 (6)	0.0175 (2)	
H11	0.3700	0.8880	0.7887	0.021*	
C12	0.33593 (16)	0.81603 (11)	0.88393 (7)	0.0180 (2)	
H12	0.4448	0.7784	0.8898	0.022*	
C13	0.07979 (15)	0.85759 (10)	0.92474 (6)	0.0158 (2)	
C14	−0.02935 (16)	0.84226 (11)	0.97723 (6)	0.0181 (2)	
H14	0.0063	0.7951	1.0169	0.022*	
C15	−0.18652 (16)	0.89577 (11)	0.97044 (6)	0.0186 (2)	
C16	−0.24385 (16)	0.96705 (11)	0.91269 (7)	0.0197 (3)	
H16	−0.3530	1.0041	0.9095	0.024*	
C17	−0.14015 (16)	0.98214 (11)	0.86128 (7)	0.0187 (2)	
H17	−0.1785	1.0297	0.8221	0.022*	
C18	0.02362 (15)	0.92796 (10)	0.86552 (6)	0.0158 (2)	
O1W	0.84280 (13)	1.08318 (9)	0.56043 (6)	0.0273 (2)	
H1W	0.7463 (16)	1.0890 (16)	0.5755 (10)	0.041*	
H2W	0.862 (2)	1.0129 (9)	0.5534 (10)	0.041*	
O2W	0.41907 (12)	0.64316 (8)	1.02803 (5)	0.02027 (19)	
H3W	0.3515 (19)	0.6916 (13)	1.0055 (8)	0.030*	
H4W	0.5009 (17)	0.6346 (15)	1.0038 (8)	0.030*	

### Atomic displacement parameters (Å^2^)

**Table:**

	*U*^11^	*U*^22^	*U*^33^	*U*^12^	*U*^13^	*U*^23^
Cl1	0.02726 (18)	0.02886 (19)	0.0343 (2)	−0.01172 (13)	−0.00194 (14)	−0.00641 (14)
Cl2	0.02222 (16)	0.03008 (18)	0.02149 (16)	−0.00523 (12)	0.00896 (12)	−0.00083 (12)
S1	0.01414 (15)	0.02233 (16)	0.01838 (16)	0.00074 (11)	0.00224 (11)	0.00574 (11)
N1	0.0181 (5)	0.0184 (5)	0.0162 (5)	0.0031 (4)	0.0028 (4)	0.0020 (4)
N2	0.0176 (5)	0.0164 (5)	0.0184 (5)	−0.0002 (4)	−0.0002 (4)	0.0014 (4)
C1	0.0206 (6)	0.0213 (6)	0.0146 (5)	0.0025 (5)	0.0017 (4)	−0.0001 (5)
C2	0.0175 (6)	0.0192 (6)	0.0179 (6)	−0.0017 (5)	−0.0014 (5)	0.0005 (5)
C3	0.0156 (5)	0.0166 (6)	0.0166 (6)	0.0013 (4)	0.0024 (4)	0.0042 (4)
C4	0.0164 (5)	0.0134 (5)	0.0159 (5)	0.0015 (4)	0.0017 (4)	0.0028 (4)
C5	0.0215 (6)	0.0171 (6)	0.0163 (6)	0.0009 (5)	0.0034 (5)	0.0012 (5)
C6	0.0262 (6)	0.0163 (6)	0.0176 (6)	0.0006 (5)	0.0008 (5)	−0.0004 (5)
C7	0.0189 (6)	0.0151 (6)	0.0244 (6)	−0.0022 (5)	−0.0028 (5)	0.0009 (5)
C8	0.0172 (6)	0.0165 (6)	0.0219 (6)	0.0005 (5)	0.0026 (5)	0.0038 (5)
C9	0.0171 (5)	0.0141 (5)	0.0154 (5)	0.0025 (4)	0.0014 (4)	0.0029 (4)
C10	0.0168 (5)	0.0136 (5)	0.0163 (5)	−0.0020 (4)	0.0002 (4)	0.0003 (4)
C11	0.0162 (6)	0.0187 (6)	0.0180 (6)	−0.0006 (5)	0.0033 (4)	0.0000 (5)
C12	0.0157 (6)	0.0169 (6)	0.0209 (6)	0.0009 (4)	0.0007 (4)	0.0002 (5)
C13	0.0165 (6)	0.0139 (5)	0.0166 (6)	−0.0027 (4)	0.0007 (4)	−0.0018 (4)
C14	0.0210 (6)	0.0163 (6)	0.0165 (6)	−0.0040 (5)	0.0011 (5)	0.0001 (4)
C15	0.0195 (6)	0.0196 (6)	0.0177 (6)	−0.0060 (5)	0.0056 (5)	−0.0033 (5)
C16	0.0165 (6)	0.0198 (6)	0.0228 (6)	0.0000 (5)	0.0027 (5)	−0.0020 (5)
C17	0.0177 (6)	0.0180 (6)	0.0201 (6)	0.0003 (5)	0.0019 (5)	0.0017 (5)
C18	0.0159 (5)	0.0146 (6)	0.0166 (6)	−0.0021 (4)	0.0012 (4)	−0.0016 (4)
O1W	0.0249 (5)	0.0254 (5)	0.0344 (6)	−0.0013 (4)	0.0139 (4)	−0.0043 (4)
O2W	0.0209 (5)	0.0223 (5)	0.0178 (4)	0.0023 (4)	0.0029 (3)	0.0030 (4)

### Geometric parameters (Å, º)

**Table:**

Cl1—C7	1.7394 (13)	C8—C9	1.4169 (17)
Cl2—C15	1.7351 (12)	C8—H8	0.9500
S1—C10	1.7657 (12)	C10—C11	1.3745 (17)
S1—C3	1.7745 (13)	C10—C18	1.4289 (17)
N1—C1	1.3116 (17)	C11—C12	1.4084 (17)
N1—C9	1.3680 (16)	C11—H11	0.9500
N2—C12	1.3183 (16)	C12—H12	0.9500
N2—C13	1.3690 (16)	C13—C14	1.4150 (17)
C1—C2	1.4158 (18)	C13—C18	1.4228 (17)
C1—H1	0.9500	C14—C15	1.3675 (18)
C2—C3	1.3677 (18)	C14—H14	0.9500
C2—H2	0.9500	C15—C16	1.4097 (18)
C3—C4	1.4267 (17)	C16—C17	1.3687 (18)
C4—C5	1.4148 (17)	C16—H16	0.9500
C4—C9	1.4229 (17)	C17—C18	1.4189 (17)
C5—C6	1.3697 (18)	C17—H17	0.9500
C5—H5	0.9500	O1W—H1W	0.846 (9)
C6—C7	1.4087 (19)	O1W—H2W	0.841 (9)
C6—H6	0.9500	O2W—H3W	0.850 (9)
C7—C8	1.3643 (19)	O2W—H4W	0.844 (9)
			
C10—S1—C3	103.31 (6)	C11—C10—C18	118.85 (11)
C1—N1—C9	117.74 (11)	C11—C10—S1	124.08 (10)
C12—N2—C13	117.29 (10)	C18—C10—S1	117.02 (9)
N1—C1—C2	124.20 (12)	C10—C11—C12	119.00 (11)
N1—C1—H1	117.9	C10—C11—H11	120.5
C2—C1—H1	117.9	C12—C11—H11	120.5
C3—C2—C1	118.82 (12)	N2—C12—C11	124.59 (11)
C3—C2—H2	120.6	N2—C12—H12	117.7
C1—C2—H2	120.6	C11—C12—H12	117.7
C2—C3—C4	119.41 (11)	N2—C13—C14	117.68 (11)
C2—C3—S1	118.44 (10)	N2—C13—C18	122.93 (11)
C4—C3—S1	121.92 (9)	C14—C13—C18	119.39 (11)
C5—C4—C9	118.70 (11)	C15—C14—C13	119.52 (11)
C5—C4—C3	124.34 (11)	C15—C14—H14	120.2
C9—C4—C3	116.96 (11)	C13—C14—H14	120.2
C6—C5—C4	121.20 (12)	C14—C15—C16	122.06 (11)
C6—C5—H5	119.4	C14—C15—Cl2	119.77 (10)
C4—C5—H5	119.4	C16—C15—Cl2	118.18 (10)
C5—C6—C7	118.95 (12)	C17—C16—C15	119.09 (12)
C5—C6—H6	120.5	C17—C16—H16	120.5
C7—C6—H6	120.5	C15—C16—H16	120.5
C8—C7—C6	122.35 (12)	C16—C17—C18	121.12 (12)
C8—C7—Cl1	119.53 (10)	C16—C17—H17	119.4
C6—C7—Cl1	118.12 (10)	C18—C17—H17	119.4
C7—C8—C9	119.17 (11)	C17—C18—C13	118.82 (11)
C7—C8—H8	120.4	C17—C18—C10	123.85 (11)
C9—C8—H8	120.4	C13—C18—C10	117.33 (11)
N1—C9—C8	117.56 (11)	H1W—O1W—H2W	108.5 (17)
N1—C9—C4	122.86 (11)	H3W—O2W—H4W	105.2 (15)
C8—C9—C4	119.59 (11)		
			
C9—N1—C1—C2	−0.18 (19)	C3—S1—C10—C11	−14.13 (12)
N1—C1—C2—C3	1.1 (2)	C3—S1—C10—C18	168.56 (9)
C1—C2—C3—C4	−0.61 (18)	C18—C10—C11—C12	−0.14 (18)
C1—C2—C3—S1	174.00 (9)	S1—C10—C11—C12	−177.40 (9)
C10—S1—C3—C2	116.54 (10)	C13—N2—C12—C11	−0.28 (18)
C10—S1—C3—C4	−68.99 (11)	C10—C11—C12—N2	0.63 (19)
C2—C3—C4—C5	179.25 (12)	C12—N2—C13—C14	179.10 (11)
S1—C3—C4—C5	4.83 (17)	C12—N2—C13—C18	−0.54 (17)
C2—C3—C4—C9	−0.62 (17)	N2—C13—C14—C15	179.97 (11)
S1—C3—C4—C9	−175.03 (9)	C18—C13—C14—C15	−0.37 (18)
C9—C4—C5—C6	−2.04 (18)	C13—C14—C15—C16	−0.46 (19)
C3—C4—C5—C6	178.10 (12)	C13—C14—C15—Cl2	179.87 (9)
C4—C5—C6—C7	0.84 (19)	C14—C15—C16—C17	0.85 (19)
C5—C6—C7—C8	0.96 (19)	Cl2—C15—C16—C17	−179.47 (10)
C5—C6—C7—Cl1	−179.30 (10)	C15—C16—C17—C18	−0.39 (19)
C6—C7—C8—C9	−1.46 (19)	C16—C17—C18—C13	−0.41 (18)
Cl1—C7—C8—C9	178.80 (9)	C16—C17—C18—C10	179.01 (12)
C1—N1—C9—C8	179.02 (11)	N2—C13—C18—C17	−179.57 (11)
C1—N1—C9—C4	−1.18 (17)	C14—C13—C18—C17	0.80 (17)
C7—C8—C9—N1	180.00 (11)	N2—C13—C18—C10	0.97 (17)
C7—C8—C9—C4	0.20 (18)	C14—C13—C18—C10	−178.66 (11)
C5—C4—C9—N1	−178.29 (11)	C11—C10—C18—C17	179.98 (12)
C3—C4—C9—N1	1.58 (17)	S1—C10—C18—C17	−2.57 (16)
C5—C4—C9—C8	1.50 (17)	C11—C10—C18—C13	−0.59 (17)
C3—C4—C9—C8	−178.63 (11)	S1—C10—C18—C13	176.86 (9)

### Hydrogen-bond geometry (Å, º)

**Table:**

*D*—H···*A*	*D*—H	H···*A*	*D*···*A*	*D*—H···*A*
O1*W*—H1*W*···N1	0.85 (1)	2.02 (1)	2.8530 (15)	171 (2)
O1*W*—H2*W*···O2*W*^i^	0.84 (1)	1.94 (1)	2.7723 (14)	173 (2)
O2*W*—H3*W*···N2	0.85 (2)	2.01 (2)	2.8429 (14)	165 (1)
O2*W*—H4*W*···O1*W*^ii^	0.85 (1)	1.94 (2)	2.7683 (14)	166 (2)

Symmetry codes: (i) *x*+1/2, −*y*+3/2, *z*−1/2; (ii) −*x*+3/2, *y*−1/2, −*z*+3/2.
